# Unraveling the deadly dance: endothelial cells and neutrophils in sepsis-induced acute lung injury/acute respiratory distress syndrome

**DOI:** 10.3389/fcell.2025.1551138

**Published:** 2025-05-22

**Authors:** Xiujuan Xu, Qi Zhang, Zheng Lv, Chuji Cheng, Junjing Zha, Huaqing Shu, Hairong Xiao, Shangwen Pan

**Affiliations:** ^1^ Department of Critical Medicine, Anqing Municipal Hospital, Anqing, China; ^2^ Department of Critical Care Medicine, Union Hospital, Tongji Medical College, Huazhong University of Science and Technology, Wuhan, China; ^3^ Department of General Surgery, Anqing Municipal Hospital, Anqing, China; ^4^ Institute of Anesthesia and Critical Care Medicine, Union Hospital, Tongji Medical College, Huazhong University of Science and Technology, Wuhan, China; ^5^ Department of Anesthesiology, Union Hospital, Tongji Medical College, Huazhong University of Science and Technology, Wuhan, China; ^6^ Key Laboratory of Anesthesiology and Resuscitation, Huazhong University of Science and Technology, Ministry of Education, Wuhan, China

**Keywords:** ALI/ARDS, neutrophils, ECs, Piezo1, sepsis

## Abstract

Sepsis-induced acute lung injury (ALI) and acute respiratory distress syndrome (ARDS) are severe complications with high morbidity and mortality rates, characterized primarily by diffuse alveolar damage, endothelial dysfunction, and local inflammatory responses. Neutrophils and endothelial cells (ECs) play crucial roles in the pathogenesis and progression of these diseases. Neutrophils are important regulators of inflammation, while endothelial dysfunction exacerbates vascular permeability and the inflammatory cascade. The interaction between neutrophils and ECs is vital for the development of ALI/ARDS induced by sepsis, driving the pathological processes of inflammation and tissue damage. Despite advancements in treatment strategies such as protective mechanical ventilation and fluid management, effective methods for rapid lung tissue recovery or significant improvement in outcomes remain lacking. Therefore, we comprehensively summarize the current literature to gain deeper insights into the roles of neutrophils, ECs, and their interactions in sepsis-induced ALI/ARDS, hoping to provide critical insights into the mechanisms underlying sepsis-related ALI/ARDS and potential pathways for developing new therapeutic approaches.

## 1 Introduction

Sepsis is a syndrome that is characterized by a dysregulated host response to infection, leading to a series of physiological, pathological, and biological abnormalities (Singer et al., 2016). Although the incidence and mortality rates have declined in recent years due to global efforts, sepsis remains one of the leading causes of death among patients worldwide. Statistics indicate that in 2017, there were approximately 49 million cases of sepsis globally, with 11 million deaths associated with sepsis, accounting for 19.7% of total global mortality (Rudd et al., 2020).

As a systemic inflammatory response syndrome, acute lung injury (ALI) and acute respiratory distress syndrome (ARDS) are serious complications of sepsis, characterized by high rates of morbidity and mortality. Compared to the common types of ALI/ARDS caused by trauma, pancreatitis, or transfusion of blood products, sepsis-induced ALI/ARDS is driven by a systemic inflammatory response syndrome (SIRS) triggered by infection. This condition is characterized by a more pronounced systemic inflammatory response, accompanied by multiple organ dysfunction syndrome (MODS). In typical ALI/ARDS, the inflammatory response is primarily localized to the lungs, while sepsis-induced ALI/ARDS elicits a robust systemic immune response due to infection, resulting in more severe damage to vascular endothelial cells (ECs) and significantly increased capillary permeability. This exacerbates pulmonary edema, hypotension, and even shock. Although various treatment strategies, including protective mechanical ventilation, restrictive fluid management, and neuromuscular blockade, are widely employed for ARDS induced by sepsis, there is currently no effective method to rapidly repair damaged lung tissue or significantly improve patient prognosis.

The primary histological feature of ARDS is diffuse alveolar damage, with core pathological mechanisms including endothelial dysfunction and localized inflammatory responses (Matthay et al., 2019). Neutrophils play a critical role as key regulators of inflammation in the pathogenesis and progression of ALI/ARDS (Wang et al., 2022). The activation and dysfunction of ECs and neutrophils are not unique to sepsis-induced ALI/ARDS; they also play a crucial role in other types of ALI/ARDS. However, in sepsis-induced ALI/ARDS, the activation and functional abnormalities of ECs and neutrophils are often more pronounced due to the influence of SIRS and infection-related factors. This excessive cellular activation and inflammatory dysregulation further exacerbate lung inflammation and systemic injury, highlighting the distinctive pathological characteristics of sepsis-induced ALI/ARDS. Therefore, this article reviews the roles of ECs, neutrophils, and their interactions in sepsis-induced ALI/ARDS based on existing literature, aiming to enhance understanding of this condition and provide potential insights for exploring clinical treatment avenues.

## 2 ECs in sepsis-induced ALI/ARDS

ECs, as a monolayer barrier lining the vascular surface and directly interacting with blood, are crucial components of the pulmonary barrier function (Shao et al., 2014). They perform various essential roles, including the regulation of vascular permeability, coagulation, and transcellular transport (substance transport). Activated ECs enhance coagulation activity and increase vascular permeability, leading to capillary thrombosis and pulmonary edema (Gando et al., 2004). Furthermore, activated ECs recruit immune cells to lung tissue, participating in the early inflammatory response and exacerbating lung injury (Qian et al., 2022). During the later stages of lung injury repair, ECs promote their own regeneration and maintain vascular homeostasis by expressing various transcription factors, thereby facilitating lung tissue repair (Liu et al., 2019; Zhao et al., 2020; Zhao et al., 2024). In the following chapters, we will conduct a detailed review of the potential regulatory points of ECs in activation and repair.

### 2.1 Activation and dysfunction of ECs in sepsis-induced ALI/ARDS

EC activation refers to the process by which these cells are stimulated by various factors, including hypoxia, cytokines, chemokines, thrombin, and bacterial lipopolysaccharides (LPS), as well as through interactions with inflammatory cells (Qiao et al., 2024). ECs express various pattern recognition receptors (PRRs), such as toll-like receptors (TLRs), which can be activated by pathogen-associated molecular patterns (PAMPs) and damage-associated molecular patterns (DAMPs). Once activated, ECs release large amounts of pro-inflammatory mediators, primarily through the NF-κB signaling pathway, exacerbating the inflammatory response by amplifying immune cell recruitment and activation. Functionally, activated ECs also exhibit increased expression of adhesion molecules, which includes P-selectin, E-selectin, intercellular adhesion molecule-1 (ICAM-1), and vascular cell adhesion molecule-1 (VCAM-1). Additionally, the activation of regulators such as tumor necrosis factor receptor-associated factor 6 (TRAF-6) (Lv et al., 2018), TRIM47, and its downstream pathway, including K63-linked TRAF2 (Qian et al., 2022), further promotes lung inflammation in ALI/ARDS by activating NF-κB signaling in ECs.

Upon activation, ECs transition into a pro-inflammatory and pro-coagulant state, which fosters the onset of oxidative stress. This inflammatory response leads to structural alterations in ECs, including damage to the glycocalyx and junctional proteins, as well as EC contraction, all of which contribute to increased vascular permeability and coagulopathy. Glycocalyx damage, driven by enzymes such as heparinase, a disintegrin and metalloproteinases (ADAMs), and matrix metalloproteinases (MMPs), further exacerbates vascular permeability. Notably, the Silent Information Regulator sirtuin 1 (SIRT1) pathway has been shown to protect the endothelial glycocalyx in ALI/ARDS (Duan et al., 2023). Damage to the glycocalyx exposes adhesion molecules that facilitate interactions between blood leukocytes and ECs. In addition, activated ECs secrete cytokines and promote the formation of NETs (Duan et al., 2023), which enhance platelet adhesion and disrupt normal coagulation processes. Simultaneously, the physiological anticoagulant pathways are impaired, further aggravating coagulation dysfunction (Ito et al., 2021).

Myosin light chain kinase (MLCK), which regulates the contraction of the perijunctional actomyosin ring through phosphorylation of the myosin II regulatory light chain, is a key regulator of tight junction permeability (Cunningham and Turner, 2012). The activation of MLCK is also implicated in thrombosis formation and coagulopathy. Specifically, thrombin activates MLCK in a Src-dependent manner, enhancing EC permeability through cell contraction (Kempf et al., 2022).

Oxidative stress is a crucial role in various cellular functions; however, excessive production of ROS can lead to EC dysfunction and death (Jiang et al., 2020). Extensive EC death is a significant contributor to the pathological progression of ALI/ARDS. Interactions with various blood cells in the bloodstream and responses to cytokines via EC surface receptors further contribute to EC death and the exacerbation of inflammation. Notably, intervention targeting the generation of NETs has been shown to reduce the severity of ferroptosis and pulmonary vascular permeability through the SDC-1/HS/HGF/cMET pathway (Huang et al., 2023). Additionally, low vascular endothelial growth factor (VEGF) levels in lung from ARDS patients have been associated with higher rates of endothelial apoptosis (Abadie et al., 2005). LPS can activate monocytes to induce apoptosis in ECs through both TNF-α-dependent and TNF-α-independent mechanisms (Stefanec, 2000). However, few therapies currently available in clinical practice are capable of effectively reducing endothelial apoptosis at the appropriate stage of disease or as a single-agent treatment in sepsis. Therefore, targeting multiple pathways and implementing timely and effective interventions to block extensive EC death may help contain disease progression. In sepsis-induced ALI, lactic acidemia promotes the release of macrophage-derived extracellular cold-inducible RNA-binding protein (eCIRP), which activates cell death pathways in pulmonary vascular ECs, driving sepsis-related pulmonary complications (Gong et al., 2024; Gong et al., 2024).

### 2.2 Endothelial regeneration and vascular repair in sepsis-induced ALI/ARDS

Endothelial barrier dysfunction is one of the core factors contributing to the poor prognosis of ALI/ARDS (Tang et al., 2024). The regeneration of ECs and subsequent vascular repair are vital processes in the recovery from these conditions (Huang et al., 2023). This repair process is crucial for maintaining vascular homeostasis and reconstructing the vascular endothelial barrier, involving the participation of many key regulatory factors.

Sox17 is an important regulatory factor in lung ECs regeneration. In LPS-induced lung injury model, Sox17 promotes ECs proliferation by upregulating the expression of Cyclin E1 (Liu et al., 2019). Another key signaling pathway involved in endothelial regeneration is the p110γPI3K signaling pathway, which is essential for activating the reparative transcription factor Forkhead box M1 (FoxM1). Studies have shown that the activation of p110γPI3K is crucial for endothelial regeneration and vascular repair after inflammatory vascular injury. Inhibition of this pathway leads to prolonged lung inflammation and increased vascular permeability, indicating that targeting p110γPI3K maybe a promising therapeutic strategy for enhancing endothelial repair in sepsis-induced ALI/ARDS (Huang et al., 2016). Likewise, the EC regeneration and vascular repair mediated by FoxM1 is considered one of the potential therapeutic pathways for sepsis-induced ARDS (Huang et al., 2023). Upstream of FoxM1, the transcription factor HIF-1α (hypoxia-inducible factor 1-alpha) plays a pivotal role in ECs-mediated vascular repair and inflammation resolution, making it another attractive therapeutic target for sepsis-induced ARDS (Huang et al., 2019). Another important aspect of endothelial repair involves endothelial progenitor cells (EPCs), which are essential for vascular repair and regeneration. Evidence suggests that therapies aimed at increasing the quantity and functionality of EPCs can significantly alleviate lung injury. For example, the administration of Rev-D4F, a high-density lipoprotein mimetic, has been shown to enhance EPC function and numbers, thereby reducing inflammation and promoting vascular repair in LPS-induced ALI models (Yang et al., 2019). Similarly, EPC transplantation could ameliorate ventilator-induced lung injury by improving epithelial permeability and reducing inflammation and apoptosis (Ju et al., 2019). Furthermore, the role of metabolic signaling in ECs is gaining attention as a promising target for therapy. Metabolic pathways in ECs are necessary for both the injury and repair phases of ALI/ARDS. During the repair phase, EC proliferation and junction reannealing are crucial for restoring vascular integrity. Targeting these metabolic pathways could provide novel therapeutic avenues for enhancing endothelial repair and improving outcomes in sepsis-induced ALI/ARDS (Gould et al., 2024). In conclusion, understanding the molecular mechanisms underlying endothelial regeneration and vascular repair in sepsis-induced ALI/ARDS is essential for developing effective therapeutic strategies. Targeting pathways such as p110γPI3K, enhancing EPC function, modulating ECs metabolism, and focusing on transcriptional regulators like Sox17 and HIF-1α represent promising approaches to promote endothelial repair and improve patient outcomes in these critical conditions.

### 2.3 Pulmonary endothelial heterogeneity—emerging EC subpopulations and their potential roles in sepsis-induced ALI/ARDS

The lung is a complex organ composed of various cell types, with ECs forming a crucial component of the pulmonary vasculature and exhibiting significant heterogeneity. This heterogeneity plays a key role in maintaining normal lung physiology, adapting to environmental changes, and responding to pathophysiological stimuli. During sepsis-induced ALI/ARDS, pulmonary ECs undergo significant alterations. Recent studies have further revealed the diversity of lung ECs, identifying multiple distinct EC subpopulations that may play specific functional roles in the development and progression of sepsis-related ALI/ARDS.

Using single-cell RNA sequencing (scRNA-seq), we have gained a deeper understanding of the heterogeneity of lung microvascular ECs. This research revealed two distinct capillary EC subpopulations: alveolar capillary endothelial cells (aCAPs) and general capillary endothelial cells (gCAPs) (Gillich et al., 2020; Schupp et al., 2021). aCAPs are a lung-specific EC subpopulation primarily responsible for gas exchange and leukocyte trafficking. This subpopulation uniquely expresses carbonic anhydrase 4 (Car4), with its expression dependent on VEGFA secreted by type I alveolar epithelial cells, and constitutes approximately 15% of the total pulmonary ECs (Vila et al., 2020). The characteristics of aCAPs are significantly affected when epithelial cells lose VEGFA; despite the structural integrity of myofibroblasts being maintained, abnormal enlargement of alveolar spaces leads to impaired lung function (Vila et al., 2020).

Unlike aCAPs, gCAPs primarily participate in the regulation of vascular tone and act as important stem/progenitor cells, maintaining and repairing capillary homeostasis. Through scRNA-seq, Seurat clustering, and differential gene expression analysis, gCAPs were further divided into two main subpopulations: immune-related ECs and development-related ECs. Immune-related ECs highly expressed immune-related genes (e.g., CD74), while development-related ECs were enriched in genes associated with angiogenesis (e.g., Sox17). Studies have report significant differences in gene expression between these two subpopulations at different stages of the inflammatory response, a phenomenon observed in multiple lung injury models (Zhang et al., 2022). During the early stage of inflammation, the expression levels of development-related genes significantly decrease. However, as inflammation resolves and tissue repair commences, these genes gradually return to high levels of expression, promoting EC regeneration (Zhang et al., 2022; Godoy et al., 2023). Furthermore, a novel proliferative EC subpopulation (proECs) was identified during the inflammatory repair phase. This subpopulation is enriched in genes associated with chromosome segregation, mitosis, DNA replication, and the cell cycle, suggesting a crucial role in endothelial regeneration (Liu et al., 2019).

Sox17 is as a key regulator of endothelial regeneration and plays a crucial role in endothelial repair following inflammatory vascular injury. Studies have shown that Sox17 overexpression not only accelerates EC regeneration but also inhibits the expression of pro-inflammatory cytokines, demonstrating its anti-inflammatory potential in the repair process (Zhang et al., 2022). However, the functional division of labor among different pulmonary vascular EC subpopulations during lung injury and repair remains incompletely understood.

In other pathological conditions, a review from Southern Medical University details the updated classification of endothelial dysfunction associated with pulmonary arterial hypertension (PAH) (Shen et al., 2024). Furthermore, research has identified another EC subpopulation closely related to pro-fibrotic processes, characterized by the expression of Cxcl12 (Liu et al., 2021).

In conclusion, the heterogeneity of pulmonary ECs offers novel therapeutic targets for ALI and ARDS. Future studies may further elucidate the functions of different EC subpopulations and their specific roles in inflammation and repair. Targeting the regulation of specific EC subpopulations and their unique responses to inflammation and mechanical stimuli holds promise as a potential strategy for treating these severe lung diseases.

## 3 Neutrophils in sepsis-induced ALI/ARDS

Neutrophils play a pivotal role in the pathogenesis of sepsis-induced ALI/ARDS. These cells are among the first responders to infection and are crucial for the innate immune response. However, their dysregulated activity can lead to significant tissue damage and exacerbate the condition of ALI/ARDS. The recruitment and activation of neutrophils in the lungs are critical processes that contribute to the progression of these conditions. In sepsis, neutrophils are often found to be dysfunctional, which can lead to impaired migration and excessive accumulation in the lungs, resulting in tissue damage and inflammation (Xu et al., 2024).

### 3.1 The role of neutrophils in early lung injury

Neutrophils play a pivotal role in the pathogenesis of early sepsis-induced ALI and ARDS. As first responders to infection, these immune cells are essential to the body’s defense mechanisms. They contribute to pathogen elimination through the production of antimicrobial peptides, ROS, and inflammatory mediators. However, their dysregulated activation and excessive accumulation in the lungs can exacerbate tissue damage and amplify inflammation, driving the progression of ALI/ARDS. In the early stages of these conditions, neutrophils act as key mediators, striking a delicate balance between protective immune responses and harmful tissue injury, ultimately influencing disease outcomes (Wang et al., 2022).

ARDS is a highly inflammatory disease strongly associated with neutrophil activation. Excessive neutrophil recruitment is a key feature of ARDS, and the released immunomodulatory molecules from these neutrophils induce tissue damage. Studies indicate that inhibiting excessive neutrophil migration or activation can significantly reduce lung injury (Conrad et al., 2022). In the context of sepsis, neutrophils are extensively activated and recruited to the lungs, releasing various cytotoxic substances, including ROS, proteases, and NETs. While intended to combat pathogens, these substances can also damage the alveolar-capillary barrier, leading to increased permeability, edema, and impaired gas exchange, all hallmarks of ALI/ARDS. Furthermore, neutrophil-derived antimicrobial peptides (such as the associated peptide LL-37), in synergy with myeloperoxidase (MPO) and histones, exert significant cytotoxicity on ECs, further exacerbating alveolar and capillary damage, ultimately leading to the development of ALI and ARDS.

Neutrophil recruitment and activation are primarily mediated by various chemokines and cytokines, such as IL-8 and CXCL1. Sustained excessive secretion of these chemokines can trigger an overzealous neutrophil response, leading to lung tissue damage. Furthermore, the formation of NETs, a process known as NETosis, is considered a “double-edged sword” in sepsis-induced ALI/ARDS. While NETs effectively trap and kill pathogens, their excessive production can cause endothelial damage and exacerbate pulmonary inflammation. Research suggests that targeting NET formation or accelerating its clearance may be an effective strategy for mitigating sepsis-induced ALI/ARDS. In ARDS patients, prolonged neutrophil lifespan further amplifies the inflammatory response, whereas promoting neutrophil apoptosis can reduce excessive NET formation and suppress inflammatory processes, thereby alleviating lung injury (Song et al., 2022; Xu et al., 2024). Excessive NET release and impaired clearance are considered significant drivers of persistent inflammation in ARDS (Gregoire et al., 2018). Furthermore, neutrophil-derived exosomal miR-30d-5p activates the NF-κB signaling pathway, inducing M1 macrophage polarization, which in turn triggers pyroptosis and exacerbates the pathological process of sepsis-induced acute lung injury (Jiao et al., 2021).

Neutrophils significantly contribute to sepsis-induced pulmonary microcirculatory dysfunction. During sepsis, excessive neutrophil accumulation in capillaries can lead to localized microcirculatory disturbances, increasing functional dead space and subsequently causing hypoxemia and pulmonary edema. This disruption of pulmonary microcirculation is a crucial factor in hypoxemia associated with ALI and ARDS. Targeting neutrophils to restore microcirculatory function has demonstrated effectiveness in alleviating hypoxemia and edema, offering a novel research avenue for sepsis-induced ALI/ARDS (Park et al., 2019).

Furthermore, the interplay between neutrophils and other immune cells, such as macrophages and T cells, plays a crucial role in modulating inflammation. These interactions can both amplify inflammatory cascades and, under specific circumstances, promote inflammation resolution (Wang et al., 2022). Investigating the mechanisms of these cellular and molecular interactions is essential for developing targeted therapeutic strategies for sepsis-inducedALI/ARDS.

In summary, while neutrophils are crucial in the initial immune response to sepsis, their dysregulated activity often triggers severe lung injury in ALI/ARDS. Modulating neutrophil function, limiting their overactivation, and targeting related signaling pathways may not only alleviate lung injury but also potentially improve the prognosis of sepsis-induced ALI/ARDS, offering potential therapeutic strategies for these conditions.

### 3.2 The role of neutrophils in late-stage and lung repair of sepsis-induced ALI/ARDS

Neutrophils participate not only in the early stages of ALI/ARDS but also in the later stages. In these diseases, neutrophils can exacerbate lung injury and initiate repair processes by participating in the clearance of apoptotic cells and debris, resolving inflammation, and restoring tissue homeostasis (Bhatia et al., 2012). First, some molecules can regulate the transition from the initial pro-inflammatory response to an anti-inflammatory state, thereby limiting further tissue damage. For example, neutrophils synthesize and release pro-resolution lipid mediators, including lipoxin A4, resolvin, and protectin, which play a critical role in alleviating lung inflammation (Serhan et al., 2008). Additionally, NETs contribute to the resolution and alleviation of inflammation by degrading excessive cytokines and chemokines (Schauer et al., 2014).

In the late stages of sepsis-induced ALI/ARDS, neutrophils exhibit altered functions, contributing to persistent inflammation and tissue damage. Impaired clearance of apoptotic neutrophils, leading to prolonged inflammation and delayed resolution of lung injury, is partly responsible. Furthermore, NETs, extracellular networks of DNA and antimicrobial proteins, further damage lung tissue by promoting endothelial and epithelial cell death and increasing vascular permeability (Fox et al., 2013; Jin et al., 2023). Dysregulation of neutrophil chemotaxis and migration is another crucial factor in the late phase of sepsis-induced ALI/ARDS. Despite high levels of chemoattractants at the site of infection, neutrophils often fail to migrate effectively, causing them to accumulate in the lungs and release harmful substances, resulting in tissue injury. This impaired migration is associated with dysregulation of signaling pathways, including those involving G protein-coupled receptors (GPCRs), which are critical for neutrophil chemotaxis (Park et al., 2019; Wang et al., 2023).

Furthermore, certain molecules released by neutrophils can directly drive tissue remodeling and repair. For example, MMPs can degrade collagen scars and inflammatory mediators, thereby promoting cell migration and epithelial regeneration (Greenlee et al., 2007; Davey et al., 2011). Furthermore, neutrophils can promote epithelial cell proliferation and repair by activating the Wnt/β-catenin signaling pathway, thereby aiding in the restoration of lung tissue function (Zemans et al., 2011). Neutrophils also actively release growth factors. These factors stimulate the proliferation and migration of epithelial and ECs, promoting the restoration of the alveolar-capillary barrier. VEGF plays a particularly important role in enhancing angiogenesis, which is crucial for regenerating a complete pulmonary microvascular network. Neutrophils can modulate tissue repair by secreting specialized pro-resolution mediators. In addition to lipid mediators like resolvins, they release cytokines and chemokines in a context-dependent manner, transitioning from a pro-inflammatory to a pro-resolving phenotype. For example, TGF-β and amphiregulin released by neutrophils can promote extracellular matrix remodeling and epithelial cell proliferation, thereby repairing damaged alveolar structures. However, excessive TGF-β may lead to aberrant repair and fibrosis, highlighting the need to balance neutrophil activity during the resolution phase.

Upon stimulation by extracellular signals such as angiotensin II or TGF-β, neutrophils can polarize and produce two distinct functional phenotypes (N1 and N2). The N1 phenotype is pro-inflammatory functions and enhanced cytotoxicity, while the N2 phenotype exhibits anti-inflammatory properties and can secrete growth factors and MMPs (such as VEGF and MMP-9), facilitating tissue repair and reconstruction (Fridlender et al., 2009). The involvement of these molecules is crucial for the regeneration of tissue following lung injury.

Moreover, neutrophils can indirectly promote lung repair processes by modulating other immune cells. An effective resolution of the inflammatory response requires macrophages to clear senescent or apoptotic neutrophils. During this process, the macrophage phenotype transitions from one that secretes pro-inflammatory cytokines and chemokines to one that releases anti-inflammatory factors, thus supporting tissue remodeling and repair (Lumbroso et al., 2018).

In summary, while neutrophils exert destructive effects during acute lung injury and inflammation, they also play a crucial role in tissue regeneration and functional recovery through reparative mechanisms, offering new avenues for lung injury treatment research. In the late stages of ARDS, damaged alveoli attempt to restore tissue homeostasis by supporting the reconstruction of alveolar structures, a period characterized by fibroblast proliferation. Neutrophils exacerbate fibrosis by releasing elastase (Takemasa et al., 2012), and NET (Chrysanthopoulou et al., 2014).

### 3.3 The role of neutrophil death mechanisms in sepsis-induced ALI/ARDS

Neutrophils play a crucial role in the immune response during sepsis, and their different modes of cell death can significantly impact the progression and severity of sepsis-induced ALI/ARDS. In sepsis, neutrophils can undergo various forms of cell death, including apoptosis, necroptosis, and pyroptosis, each of which has distinct implications for inflammation and tissue damage.

The inflammatory response is a fundamental physiological and pathological process, with the inflammasome serving as a key regulator. Inflammasomes recruit and activate caspase-1, which not only promotes the hydrolytic maturation of IL-1β and IL-18 but also triggers the cleavage of Gasdermin D (GSDMD). The cleaved GSDMD forms pores in the cell membrane, initiating pyroptosis (Broz et al., 2020). During this process, the release of IL-1β, IL-18, and HMGB1 exacerbates local inflammation, causes tissue damage, and transmits danger signals from damaged or dying cells to the surrounding environment. This cascade further stimulates immune cell activity, particularly neutrophil recruitment. While intracellular pathogens are not directly released into the extracellular space during neutrophil pyroptosis, they can form pore-induced intracellular traps. This mechanism, in conjunction with the complement system and clearance receptors, enhances the innate immune response by recruiting neutrophils, which subsequently release ROS or engage in secondary phagocytosis to eliminate pathogens. Notably, neutrophil pyroptosis also serves as a trigger for the formation of NETs.

Necroptosis, a form of programmed necrosis, triggers the release of cellular contents, exacerbating inflammatory responses. Research indicates that this cell death pathway significantly worsens inflammation in sepsis-induced ALI/ARDS, leading to lung injury and dysfunction (Aziz et al., 2014). Specifically, neutrophil necroptosis is often closely associated with the formation of NETs. NET formation induces endothelial dysfunction and increases vascular permeability, thereby aggravating pathological changes (Xu et al., 2024). Furthermore, neutrophil-derived enzymes and ROS released during necroptosis further disrupt the alveolar-capillary barrier, worsening lung damage (Blazquez-Prieto et al., 2018). Notably, the role of autophagy in regulating neutrophil function, including necroptosis, has garnered attention as a potential therapeutic target to mitigate lung injury during sepsis (Park et al., 2017). In summary, neutrophil necroptosis is a crucial factor in the pathogenesis of sepsis-induced ALI/ARDS, significantly contributing to the excessive inflammation and tissue damage.

Apoptosis, a form of programmed cell death, is generally associated with anti-inflammatory effects, unlike other cell death pathways. It is characterized by the orderly dismantling of cells and their clearance by macrophages without the release of inflammatory mediators. Studies have shown that delayed neutrophil apoptosis exacerbates lung injury, while promoting timely apoptosis aids in inflammation resolution (Li et al., 2014; Park et al., 2019). The molecular mechanisms of neutrophil apoptosis are complex, involving the activation of death-associated protein kinase 1 (DAPK1) and caspases, pathways that play a central role in regulating neutrophil apoptosis and inflammation^[60–61]^. For instance, the lipid mediator protectin D1 has been shown to enhance neutrophil apoptosis, thereby accelerating inflammation resolution. Similarly, research indicates that nanoparticle-induced apoptosis can significantly improve survival rates in sepsis and effectively alleviate lung injury (Paunel-Gorgulu et al., 2012; Li et al., 2014). Furthermore, recent findings suggest that Gasdermin E (GSDME) is a crucial protein regulating the switch between apoptosis and pyroptosis (Ma et al., 2024). Through the action of specific enzymes, such as caspase-3, GSDME modulates the conversion between these cell death pathways, further influencing neutrophil fate and its role in lung inflammation. However, in sepsis, excessive apoptosis of various immune cells, such as neutrophils and macrophages, can lead to immunosuppression, disrupting cellular balance and increasing the risk of secondary lung injury.

In conclusion, the different modes of neutrophil death in sepsis-induced ARDS have profound effects on the inflammatory response and tissue damage. Targeting specific pathways involved in neutrophil death may offer novel therapeutic strategies to improve outcomes (Englert et al., 2019).

### 3.4 Heterogeneity of neutrophils in the lungs: new subpopulations and their roles in ALI/ARDS

For a long time, neutrophils have been considered key players in the pathogenesis of ALI/ARDS. However, despite significant advancements in neutrophil research, clinical outcomes for ALI/ARDS have not substantially improved. In recent years, neutrophil research has shifted from traditional functional exploration to heterogeneity analysis, revealing their complexity and diversity in disease states. In ALI/ARDS, classically defined neutrophil subtypes participate in inflammatory cascades, pathogen clearance, and tissue repair by initiating and amplifying inflammatory responses, releasing ROS and NETs, and secreting proteases and modulating immune cell interactions. However, other distinct neutrophil subsets may perform a variety of unique functions. Therefore, in-depth investigation of the diversity and functions of neutrophil subsets within the lung microenvironment is crucial for further understanding the complex mechanisms of ALI/ARDS and developing targeted therapeutic strategies.

Using single-cell RNA sequencing (scRNA-seq), researchers identified two distinct neutrophil subpopulations in the lung tissue of an ALI mouse model, namely, *F*th1^hi^ Neu and Prok2^hi^ Neu, which exhibit unique functional and distributional characteristics (Wang et al., 2022). These subpopulations can be clearly distinguished by specific gene expression patterns. *F*th1^hi^ Neu is mainly distributed within and around the airways (e.g., bronchi, alveolar lumen, and interstitial regions), morphologically tending to exhibit multilobed or even hypersegmented nuclei, and displaying aging-associated phenotypic features. Functionally, this subpopulation exhibits strong pro-inflammatory and chemotactic properties, while demonstrating reduced sensitivity to apoptosis and oxidative damage. Prok2^hi^ Neu is primarily found within lung vasculature, characterized by ring-shaped nuclei, and exhibits an immune-activated state. These neutrophils express high levels of antimicrobial peptides, exhibit active glycolysis metabolically, and undergo rapid apoptosis, thereby being promptly cleared by the immune system to avoid excessive inflammation.

In the ALI mouse model, the proportion of *F*th1^hi^ Neu neutrophils is significantly increased, and its level is positively correlated with disease severity. Furthermore, in ARDS patients, neutrophils with high *F*th1 expression and low Prok2 expression may predict poorer clinical outcomes. The study also indicates that the anti-inflammatory cytokine IL-10 can alleviate ALI-induced acute pulmonary inflammation and diffuse alveolar damage by regulating the ratio of *F*th1^hi^ Neu to Prok2^hi^ Neu subpopulations (Wang et al., 2022). These findings suggest that these specific neutrophil subtypes may serve as potential prognostic biomarkers for ARDS patients and provide new ideas for targeted therapy.

Compared to studies on pulmonary ECs, research on neutrophil heterogeneity is relatively limited. However, recent studies are gradually uncovering the diversity of neutrophils in different pathological states. For instance, Xie et al. used single-cell transcriptomic analysis to reveal the heterogeneity of neutrophils and their coordinated maturation process during homeostasis and bacterial infection (Xie et al., 2020). Furthermore, in cancer research, Liu et al. explored the origin, chemotaxis, and activation regulators of tumor-associated neutrophils, as well as their phenotypes and functions in the tumor microenvironment (Liu et al., 2023).

In conclusion, the diversification of neutrophil subsets plays a significant role in the pathogenesis of ALI/ARDS. The dynamic distribution and functional changes of these subsets offer not only new perspectives for investigating disease mechanisms but also potential applications for clinical intervention and prognostic assessment. With further research, precision therapeutic strategies targeting neutrophil heterogeneity may bring new breakthroughs in the treatment of ALI/ARDS.

## 4 EC-neutrophil interactions in sepsis-induced ALI/ARDS

Sepsis-induced ALI/ARDS are life-threatening conditions. The complex interplay between ECs and neutrophils is a critical mechanism in this pathological process. ECs form a crucial barrier between the bloodstream and lung tissue, essential for maintaining vascular integrity and regulating immune cell migration. Neutrophils, as the first line of immune defense, play a dual role in combating infection and causing tissue damage during sepsis. This dynamic interaction between these 2 cell types is both a protective mechanism against invading pathogens and a driver of inflammatory dysregulation in sepsis. Understanding these complex interactions is crucial for elucidating the molecular mechanisms of sepsis-induced ALI/ARDS and identifying potential therapeutic targets. In the following sections, we will elaborate on the interaction between ECs and neutrophils.

### 4.1 Regulation of neutrophil function by ECs

Neutrophil infiltration is a finely regulated multi-step process involving critical phases such as rolling, adhesion, crawling, and transmigration. In this process, activated ECs promote neutrophil rolling and initial adhesion on the vascular endothelium through the expression of surface adhesion molecules, including ICAM-1, VCAM-1, P-selectin, and E-selectin, thereby facilitating neutrophil migration into underlying tissues (Gerhardt and Ley, 2015). During transmigration, neutrophils are activated by chemokines released from ECs, resulting in increased expression of integrins on their surface, which form more stable adhesions with ICAM-1 and VCAM-1, initiating the process of crossing the endothelium to reach damaged tissues (Yuan et al., 2012; Zhong et al., 2018). Additionally, the glycosylated RNA on the neutrophil surface participates in its migration to sites of inflammation by binding to P-selectin on ECs (Zhang et al., 2024). Throughout the transmigration process, the mechanical squeezing force exerted by neutrophils activates signaling pathways in ECs, involving the interaction and activation of Platelet-Endothelial Cell Adhesion Molecule-1 (PECAM-1, or CD31) with the VEGFR2/VE-cadherin mechanotransduction complex in ECs. This interaction increases the intracellular calcium ion concentration in ECs, which further facilitates neutrophil passage across the vascular endothelium (Fu et al., 2023). ROS, and other oxidants generated by activated ECs contribute to tissue damage by neutralizing local antioxidants. In this process, VE-cadherin expression is downregulated, while neutrophil adhesion molecules are upregulated, with increased release of chemokines promoting neutrophil inflammatory migration (Millar et al., 2016). The activated integrin Macrophage-1 antigen (Mac-1) binds to ligands of ECs (e.g., ICAM-1) to promote neutrophil adhesion, crawling, and transmigration across the endothelium (Li et al., 2024). Under the influence of chemokines, the cytoskeleton of ECs undergoes dynamic remodeling, facilitating neutrophil passage through the vascular wall. Furthermore, the levels of Syndecan-4, a key component of the endothelial glycocalyx, are significantly elevated in sepsis-induced ALI/ARDS. Its downregulation, whether in in vivo or *in vitro* models, markedly increases neutrophil adhesion and inflammatory response (Zhu et al., 2023). Thus, ECs engage in the entire process of neutrophil extravasation through multiple mechanisms.

During neutrophil transmigration across ECs, dynamic changes in surface-associated molecules regulate their functional characteristics. Neutrophils experience mechanical forces during transmigration, which have significant implications for their functionality. Among these, the mechanosensory channel protein Piezo1 detects mechanical signals as neutrophils traverse the intercellular junctions of ECs. Activation of Piezo1 induces the upregulation of the Nox4 gene in neutrophils, resulting in increased hydrogen peroxide production, thereby enhancing their bactericidal capacity. Moreover, this shear force, via Piezo1 activation, can also induce NETs, presenting a potential target for studying inflammatory and thrombotic diseases related to NETs (Baratchi et al., 2024).

**FIGURE 1:**
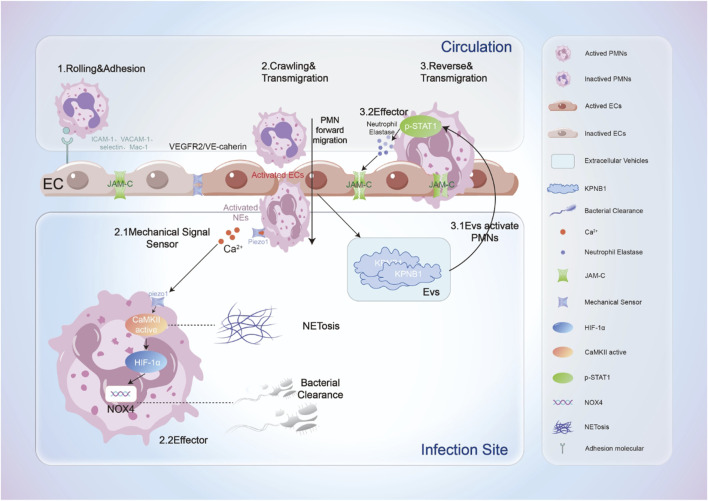
The process by which neutrophils interact dynamically with ECs when recruited to sites of inflammation. This includes: 1. Rolling and adhesion: Neutrophils roll along the surface of ECs and establish initial adhesion by binding to adhesion molecules on the endothelial surface; 2. Crawling and transmigration: ECs are activated by mechanical forces and other agents, further facilitating the transendothelial migration of neutrophils. Simultaneously, neutrophils sense mechanical stimuli, leading to the activation of their mechanosensors, which induces cell activation accompanied by an increase in intracellular calcium ion concentration. This activation results in NETosis (formation of extracellular traps) in neutrophils; on the other hand, the upregulation of Nox4 levels enhances the bactericidal capacity of neutrophils; 3. Reverse and transmigration: Activated ECs release extracellular vesicles that stimulate neutrophils to release neutrophil elastase (NE). Neutrophil elastase degrades the JAM-C connections between ECs, prompting neutrophils to undergo reverse transendothelial migration, ultimately leading to damage in distal tissues.

In addition to facilitating neutrophil migration to inflamed tissues, ECs may induce abnormal transport of neutrophils, resulting in damage to distal tissues. Inflammation-activated ECs release extracellular vehicles (EVs) rich in the transport protein subunit KPNB1, which upregulate neutrophil elastase (NE) activity via STAT signaling, leading to the degradation of junctional adhesion molecule-C (JAM-C). This promotes the reverse transport of neutrophils and induces a phenotypic shift that results in a unique form of reverse trans-endothelial migration (rTEM) activation phenotype (Zi et al., 2024). Reverse migration primarily relies on JAM-C; particularly under ischemic conditions, neutrophils expressing high levels of Mac-1 release elastase to degrade JAM-C, thereby facilitating their reverse migration (Scheiermann et al., 2009). Further studies indicate that neutrophils undergoing reverse migration predominantly transfer to the lungs, where they become sequestered, resulting in lung injury. However, the specific mechanisms underlying this process warrant further exploration. Additionally, not only can EC damage induced by pathogenic infections promote reverse migration of neutrophils, but aging ECs can also enable neutrophils to reverse migrate from localized inflammatory tissues back into the circulation, leading to distal tissue damage, particularly significant in lung tissue (Barkaway et al., 2021). This mechanism of heightened pulmonary susceptibility aligns with the clinically observed phenomenon of a significantly higher incidence of sepsis-induced ARDS compared to other organ injuries. Importantly, while reverse migration of neutrophils reduces the cellularity at the inflammatory sites, potentially mitigating the expansion of local inflammatory responses, the associated damage to distal organs cannot be overlooked. Therefore, precise modulation of neutrophil reverse migration holds considerable therapeutic significance for treating various diseases characterized primarily by neutrophil infiltration as a pathological mechanism (Nourshargh et al., 2016) (Figure 1).

### 4.2 Regulation of endothelial function by neutrophils

The endothelial barrier primarily consists of three major components: the endothelial glycocalyx, intercellular junctions, and focal adhesion on the basal side. Neutrophils profoundly regulate the integrity and function of the endothelial barrier by altering these structures and their functions. During respiratory bursts, neutrophils generate ROS, and the release of MPO, elastase, cathepsin G, and MMPs during degranulation can cleave the endothelial glycocalyx (Ma et al., 2019). Furthermore, NETs released by neutrophils accelerate the degradation of the glycocalyx (Zhang et al., 2023). Inflammatory cytokines induced by neutrophils, such as IL-6, IL-8, and TNF-α, also contribute to the disruption of glycocalyx structure, significantly increasing endothelial permeability (Schmidt et al., 2012). Once the glycocalyx is compromised, ICAM-1, E-selectin, and other adhesion molecules are exposed on the surface of ECs. This molecular exposure not only triggers a strong inflammatory response but also promotes the rolling and adhesion of leukocytes and platelets, further amplifying inflammation (Becker et al., 2010; Iba and Levy, 2019). Among the intercellular junctions, VE-cadherin is particularly susceptible to degradation by MMPs secreted by neutrophils, leading to the disruption of the endothelial barrier (Luplertlop et al., 2006; Dejana et al., 2008). Concurrently, TNF-α activates the NF-κB signaling pathway, impairing the tight junction protein claudin-5 at the endothelial junctions, further weakening the barrier function (Clark et al., 2015).

By secreting inflammatory cytokines and chemokines, neutrophils become key mediators in the regulation of endothelial function during ALI. Their derived antimicrobial peptides, such as LL-37, in conjunction with MPO and histones, exert cytotoxic damage on ECs, causing capillary injury and contributing to ALI/ARDS. In sepsis-induced ARDS, the fatty acids halogenated by myeloperoxidase released from neutrophils, specifically 2-chlorofatty acids (2-ClFAs), are closely associated with damage to pulmonary ECs (Meyer et al., 2017). Moreover, neutrophils promote the expression of tissue factor (TF) in ECs, inducing a transition to a pro-inflammatory and pro-coagulant phenotype, further compromising endothelial barrier function (Kim et al., 2016). However, it is noteworthy that during the course of sepsis, neutrophils can also mitigate oxidative stress and endothelial dysfunction by releasing extracellular vesicles carrying superoxide dismutase 2 (SOD2), thus alleviating symptoms of disseminated intravascular coagulation (DIC) (Bao et al., 2022). This protective mechanism highlights the complex role of neutrophils in endothelial barrier function, suggesting future research directions for precision therapies targeting endothelial barrier-related diseases through the modulation of neutrophil function (Figure 1).

## 5 Outlook

**FIGURE 2:**
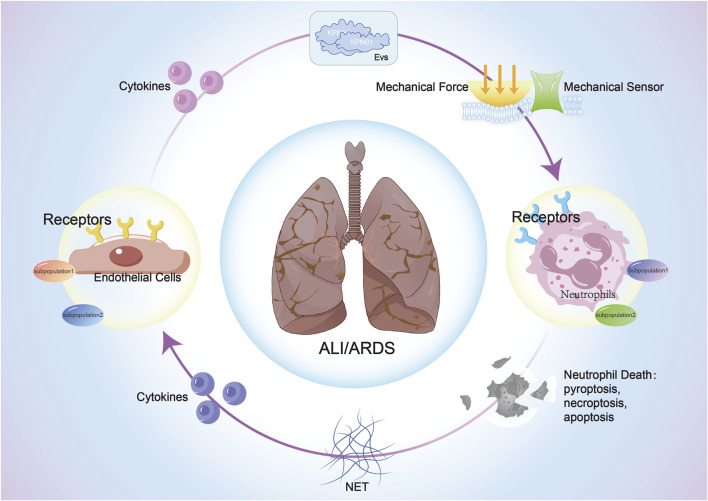
The interaction between ECs and neutrophils in sepsis-induced ALI/ARDS: Both cell types activate corresponding signaling pathways through the release of cytokines, extracellular vesicles, and mechanical force changes after stimulation, impacting cellular structure, function, phenotype, and fate, thereby influencing the progression of ALI/ARDS.

Cell-cell interactions (CCIs) are the basic mechanisms for information exchange and collaborative work between cells in multicellular organisms. Molecular interaction between cells plays the most irreplaceable part. Here, we summarize three research that may have more invaluable discoveries in neutrophil-endothelial interaction in ALI/ARDS (Figure 2).

### 5.1 The appeal of NETosis

Neutrophils play a crucial role in ALI, with NETs being a common pathogenic mechanism. The process of NET formation and release is termed NETosis. This mechanism involves the release of filamentous extracellular structures by neutrophils in response to external stimuli, capturing pathogens within a network composed of DNA, histones, proteases, and other cytotoxic and inflammatory compounds. As an important innate immune response, NETosis helps protect the body from pathogenic attacks. However, its role is not limited to pathogens; excessive or uncontrolled NETosis can cause significant damage to surrounding tissues. In sepsis-induced ALI/ARDS, the production of NETs is closely related to the severity of the disease (Xu et al., 2024). Research indicates that free DNA within NETs can lead to alveolar dysfunction and epithelial cell damage (Gan et al., 2018). NETosis markedly exacerbates EC damage, enhancing vascular leakage and aggravating sepsis-induced ARDS (Xu et al., 2024). Additionally, PAD4 (protein arginine deiminase 4) is a key enzyme involved in NET formation. Overexpression of PAD4 leads to vascular injury by releasing NETs and inducing the expression of endothelial ICAM-1 and VCAM-1. Experimental studies have shown that complete knockout of PAD4 or the use of PAD4 inhibitors can significantly suppress NET formation, thereby alleviating lung damage (Biron et al., 2018). Furthermore, histones within NETs have been found to induce apoptosis in pulmonary vascular ECs, further exacerbating sepsis-related ALI/ARDS progression (Li et al., 2021). NETosis also disrupts endothelial glycocalyx by releasing and secreting inflammatory cytokines. Simultaneously, NETs can induce macrophages M1 phenotype, leading to the release of more inflammatory cytokines and worsening the inflammatory response (Song et al., 2019). Additionally, NETs promote platelet adhesion, activation, and aggregation, providing scaffolding for thrombus formation. The interaction between platelets and ECs as well as complement further mediates NET generation, triggering immune thrombosis and creating a positive feedback loop in the coagulation process, ultimately resulting in severe endothelial dysfunction (Kumar et al., 2019; Wei et al., 2023).

In summary, in the pathological microenvironment of ALI/ARDS, NETosis significantly impacts alveolar epithelial cells, ECs, macrophages, and platelets. Therapeutic interventions targeting NETs may provide new strategies and directions for treating sepsis-induced ALI/ARDS.

### 5.2 Immune ECs (IMECs)

ECs possess extensive physiological functions, including facilitating the passage of immune cells across the endothelial barrier, regulating hemostasis and thrombosis, and achieving repair and vascular reconstruction following endothelial injury. Through single-cell transcriptomic studies, several functional subsets of ECs have been identified, one of which is enriched with immune regulation-related genes and is referred to as “immune regulatory ECs” (IMECs).

The lungs, as a highly vascularized organ, primarily consist of microvascular ECs. Because the lungs are directly connected to the external environment, they are exposed to potential pathogenic microorganisms and pollutants, with their primary function being gas exchange. However, the successful completion of this process relies on appropriate immune responses. Research has shown that under physiological conditions, IMECs are rich in MHC II-related genes, which is important in both innate and adaptive immune responses. It is hypothesized that IMECs act as “sentinels,” activating host immune defenses upon sensing airborne pathogens. Furthermore, in lung injury models induced by SARS-CoV-2 and H1N1, immune regulatory ECs exhibited a more rapid and intense inflammatory response, suggesting that the immune regulatory functions of IMECs are critical in addressing pulmonary infections and injuries.

Nevertheless, knowledge about the effects of this specific EC subset on lung injury and repair in ALI/ARDS remains limited. Exploring the immune regulatory functions of IMECs in ALI/ARDS and their contributions to pathogenesis may provide new perspectives for potential therapeutic strategies. This area holds significant frontier research importance.

### 5.3 Mechanosensors

The process by which cells sense and respond to mechanical forces, initiating biological activities, is referred to as mechanotransduction. This process has a significant impact on the development and function of organisms, with the groundbreaking discovery of Piezo channels providing a novel perspective for research in this field. Piezo proteins are mechanically sensitive ion channels that can sense biological mechanical forces such as hydrostatic pressure, fluid shear stress, and membrane stretch, transducing mechanical signals into biological signals by regulating calcium ion concentration changes. Piezo proteins are widely distributed in the heart, brain, vasculature, and musculoskeletal systems, participating in various pathophysiological processes (Jia et al., 2024; Lim et al., 2024).

Piezo1 can sense the transmigration of neutrophils across ECs, thereby enhancing their bactericidal ability and participating in immunomodulation (Mukhopadhyay et al., 2024). Concurrently, EC Piezo1 is activated by the synergy between leukocytes and the hemodynamic microenvironment, leading to downstream signaling pathways that disrupt the endothelial barrier and exacerbate leukocyte extravasation. Piezo1, expressed in ECs and type II alveolar cells, may play a detrimental role in regulating pulmonary endothelial barrier function in ventilator-induced lung injury (Friedrich et al., 2019; Liang et al., 2019). In summary, Piezo proteins play a crucial role in intercellular interactions and provide new research directions and potential therapeutic targets for various mechanotransduction-related diseases.

In the pathogenesis of ALI/ARDS, various scenarios involving neutrophils, such as their traversal through ECs, reverse migration, interactions with endothelial adhesion molecules, and the disruption and reconstruction of the endothelial barrier, all entail significant interactions with biomechanical forces. However, current research on these mechanotransduction-related mechanisms still presents substantial room for exploration, particularly regarding the role of Piezo channels in this process, which has not been thoroughly elucidated. Therefore, further investigation into the specific functions and mechanisms of Piezo channels in ALI/ARDS may bring new avenues for the diagnosis and treatment of related diseases.

## 6 Summary

In this review, we systematically explored the roles of ECs and neutrophils in sepsis-induced ALI/ARDS and their mechanisms of interaction. The interplay between ECs and neutrophils occurs through various pathways, including the recognition and binding of surface molecules, the secretion of cytokines, the release of EVs, the formation of NETs, and interactions involving mechanical forces such as membrane stretching. These interactions can either activate or inhibit corresponding signaling pathways, profoundly affecting cellular functional states and playing a critical role in the pathological progression of ALI/ARDS. These mechanisms not only provide an important basis for understanding the pathogenesis of ALI/ARDS but also indicate directions for exploring novel therapeutic strategies. A deeper understanding of the complex and nuanced interactions between ECs and neutrophils is essential for targeted therapies that regulate inflammatory responses and promote tissue repair.
